# Midline thalamic neurons are differentially engaged during hippocampus network oscillations

**DOI:** 10.1038/srep29807

**Published:** 2016-07-14

**Authors:** Ariel Lara-Vásquez, Nelson Espinosa, Ernesto Durán, Marcelo Stockle, Pablo Fuentealba

**Affiliations:** 1 Laboratorio de Circuitos Neuronales, Departamento de Psiquiatria, Centro Interdisciplinario de Neurociencia, Pontificia Universidad Catolica de Chile, Marcoleta 391, 8330024 Santiago, Chile

## Abstract

The midline thalamus is reciprocally connected with the medial temporal lobe, where neural circuitry essential for spatial navigation and memory formation resides. Yet, little information is available on the dynamic relationship between activity patterns in the midline thalamus and medial temporal lobe. Here, we report on the functional heterogeneity of anatomically-identified thalamic neurons and the differential modulation of their activity with respect to dorsal hippocampal rhythms in the anesthetized mouse. Midline thalamic neurons expressing the calcium-binding protein calretinin, irrespective of their selective co-expression of calbindin, discharged at overall low levels, did not increase their activity during hippocampal theta oscillations, and their firing rates were inhibited during hippocampal sharp wave-ripples. Conversely, thalamic neurons lacking calretinin discharged at higher rates, increased their activity during hippocampal theta waves, but remained unaffected during sharp wave-ripples. Our results indicate that the midline thalamic system comprises at least two different classes of thalamic projection neuron, which can be partly defined by their differential engagement by hippocampal pathways during specific network oscillations that accompany distinct behavioral contexts. Thus, different midline thalamic neuronal populations might be selectively recruited to support distinct stages of memory processing, consistent with the thalamus being pivotal in the dialogue of cortical circuits.

The thalamus is a heterogeneous region with topographical projections covering most of the cortical mantle^1^. Historically, lateral thalamic nuclei and their sensorimotor function have been studied in great detail^1^,^2^,^3^. Conversely, less explored is the so-called “limbic thalamus”, comprising structures located along the midline; including medial, anterior, intralaminar, and midline nuclei^4^. These structures encompass a narrow band of small nuclei distributed over the entire dorso-ventral extension of the thalamus^5^. A particular anatomical feature of the midline thalamus is its enriched expression of calcium-binding proteins. Indeed, mapping studies have revealed abundant calretinin (CR)- and calbindin (CB)-expressing somata along the midline thalamus^6^,^7^,^8^. These proteins are fundamental to regulate Ca^2+^ homeostasis and are implicated in the fine control and timing of synaptic Ca^2+^ signals^9^,^10^. In several brain regions, particularly the neocortex and hippocampus, CB and CR are robust molecular markers of discrete GABAergic neuronal populations^11^,^12^,^13^,^14^. However, similar insight has not been provided for the thalamus, where it is currently not known if calcium-binding proteins, such as CR or CB, can dissect specific cell types.

On the other hand, the functional role of the midline thalamus is just beginning to be understood. Initially, it was proposed that the main role of the midline thalamus is to adjust activity levels in target structures^5^. Nonetheless, in recent years insight has emerged on the relevance of several midline thalamic nuclei in regulating cognitive function. Indeed, it is now known that the paraventricular nucleus, part of the dorsal midline thalamic group, is necessary for fear memory^15^. In addition, the reuniens nucleus, from the ventral midline thalamic group, is required for some forms of goal-directed behaviour^16^ and memory specificity^17^. Moreover, the reuniens nucleus is implicated in system consolidation of memory^18^,^19^ and strategy shifting^20^, and recent experiments have demonstrated that is contains head-direction cells^21^. Such cognitive and executive functions are implemented by the coordinated action of the frontal and medial temporal lobes^22^,^23^; suggesting that the midline thalamus is probably important to sustain cortical interactions underlying memory processing^5^,^24^,^25^. In fact, the hippocampus is directly targeted by ventral midline thalamic nuclei, particularly by the reuniens and rhomboidal nuclei^26^,^27^,^28^, whereas most of the midline thalamus receives reciprocal afferents from the hippocampus via the subiculum^29^,^30^,^31^,^32^. These anatomical and physiological evidence support the idea that the midline thalamus contributes to the coordination of temporal and frontal lobes in the implementation of cognitive function^4^.

The hippocampus constitutes a central hub of the medial temporal lobe, which synchronizes neuronal activity along the temporal cortical axis, and also establishes coherent activity with the frontal lobe, which is required for learning and memory^33^,^34^,^35^. Two very characteristic oscillatory patterns of activity are present in the hippocampus, namely theta oscillations and sharp wave-ripples, which define particular brain states in behaving animals^33^. Each of these activity patterns seems to be associated with a specific phase of memory formation. Accordingly, theta waves (4–8 Hz oscillations in the local field potential) are prominent during exploratory states, and have been proposed to reflect memory encoding^36^; whereas ripple episodes (100–200 Hz oscillations in the local field potential) take place during quiet states (including slow wave sleep) and are believed to participate selectively in memory consolidation^37^. Given the global cortical effects of both theta and ripple oscillations, and the anatomical pathways linking the hippocampus and midline thalamus (i.e., subiculum and entorhinal cortex^4^,^27^,^29^,^31^,^32^), it becomes relevant to account for the functional connectivity between these networks during oscillatory activity.

Accordingly, here we recorded single-neuron and network activity in identified thalamic nuclei during hippocampal network oscillations. Our results indicate that the midline thalamic system comprises at least two sets of thalamic projection neuron defined by the expression of calcium-binding proteins, which are differentially engaged by hippocampal pathways during distinct network oscillations. These data establish a previously unidentified functional connection between the midline thalamus and hippocampus. Thus, diverse midline thalamic neuronal populations might be selectively recruited to support particular stages of memory processing.

## Results

### Calretinin and calbindin expression in midline thalamic neurons

**Figure 1:**
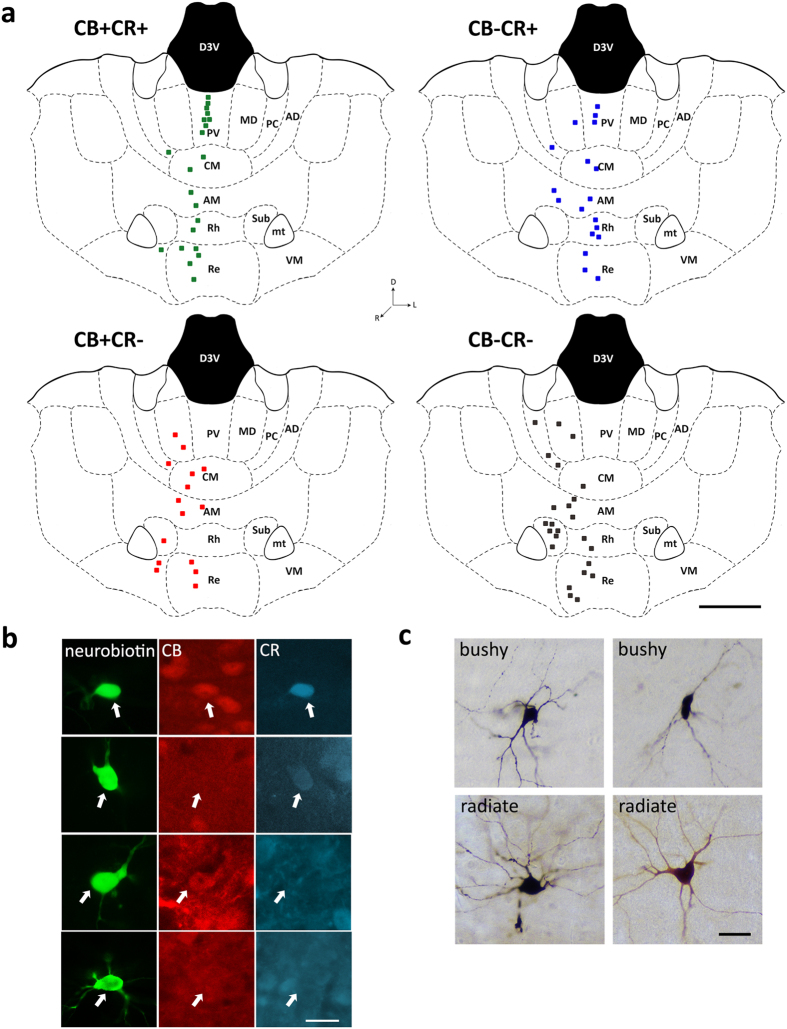
Anatomical location, neurochemical profile, and somatodendritic distribution of recorded midline thalamic neurons. (**a**) Coronal views of four identical plates containing the sample of identified neurons (n = 79), separated according to the expression of CB and CR (plus (+) and minus (−) symbols depict immunopositive and immunonegative cells, respectively). Shown is the anatomical location and molecular markers expressed in midline thalamic neurons based on the mouse brain atlas (adapted from plate 39^39^). CR+CB+, n = 21 cells; CR+CB−, n = 18 cells; CR−CB+, n = 15 cells; CR−CB−, n = 25 cells. One labelled cell (ED14c2, recorded in PT; CB−CR−) is not shown in this figure for it was located in distant anteroposterior coordinates (plate 34). AD, anterodorsal nucleus; AM, anteromedial nucleus; CM, centromedial nucleus; MD, mediodorsal nucleus; mt, mammillothalamic tract; PC, paracentral nucleus; PT, paratenial nucleus; PV, paraventricular nucleus; Re, reuniens nucleus; Rh, rhomboidal nucleus; Sub, submedial nucleus; VM, ventromedial nucleus. (**b**) Examples of diverse neurochemical profiles for the recorded and labelled thalamic neurons (arrows). Note top cell is CB+CR− (RC20c3, recorded in CM), middle cells are CB−CR+ (AL58c3, recorded in PV) and CB+CR+ (AL63c2, recorded in VM), bottom cell is CB−CR− (RC20c2, recorded in Sub). (**c**) Examples of somatodendritic distributions of DAB horseradish peroxidase product-labeled thalamic cells that were recorded and labelled *in vivo* (Supplementary Figure 2). Top panels; examples of “bushy” cells (left; AL53c7, recorded in AM; and right; AL35c2, recorded in MD). Bottom panels, examples of “radiate” cells (left; AL19c1, recorded in AM; and right; ED01c3, recorded in Re). Scale bars: (**a**) 1 mm; (**b,c**) 25 μm.

We sampled the action potential firing of individual neurons from thalamic nuclei across the midline (Table 1). After recording, neurons were individually labeled with the juxtacellular filling method^38^ and classified by their expression of neurochemical markers (Supplementary Table 1). In order to map the location of recorded neurons on the thalamus, we projected all labelled neurons on the neuroanatomical atlas of the mouse brain^39^. Neurons were recovered from most midline thalamic nuclei (Fig. 1a). The somatodendritic distributions of midline thalamic neurons were relatively uniform, with no obvious distinct morphological types. However, we could identify cells conforming to the distinct branching patterns of dendrites described in thalamic relay cells, such as “bushy” and “radiate” neurons^1^,^40^. Indeed, some cells had more angular and multipolar cell bodies, with dendritic trees branching dichotomously, characteristic of “radiate” cells (Fig. 1c). Conversely, other cells had elongated somata, with dendrites longer and branching less frequently, particularly in regions proximal to the soma, and followed a “bushy” organization (Fig. 1c). We were not able to fill the entire extension of axonal arborizations, even in cases where intense somatic labeling was achieved. In fact, most labelled cells did not show evidence of axonal labelling. When labelled, axons arose in all cases from the soma and rarely branched locally (Supplementary Figure 1). Given the above mentioned dendritic branching patterns, as well as axonal branching patterns, with little or no local collaterals, we considered our sample to be entirely comprised of thalamic projection neurons, which are known to be glutamatergic and typically reach the cortical mantle^1^. In order to characterize the neurochemical profiles of thalamic neurons, we tested the neurobiotin-labelled cells for expression of immunoreactivity for two calcium-binding proteins that have been reported to be abundantly expressed in the midline thalamus namely, calretinin (CR) and calbindin (CB)^6^,^7^,^8^. Nearly half of the labelled neurons expressed calbindin (CB+, 45.6%, n = 36) or calretinin (CR+, 49.4%, n = 39), showing that such neurochemical markers detect a large fraction of midline thalamic neurons (Supplementary Table 1). Neurons expressing combinations of CB and CR could be found in most nuclei across the midline thalamus (Fig. 1, Table 1). The expression of CR and CB largely overlapped in our neuronal sample. Indeed, nearly half of labelled neurons co-expressed both markers (47.7%, Table 1). Furthermore, neither the expression of CB nor CR was associated with distinct morphological somatodendritic distributions (i.e., “radiate” or “bushy”) in thalamic midline neurons (Supplementary Figure 2). Hence, both proteins CB and CR were widely represented in the midline thalamus, and their combinatorial expression defined four neurochemical populations (Table 1).

### Calretinin-expressing neurons exhibit low levels of spontaneous activity

**Figure 2:**
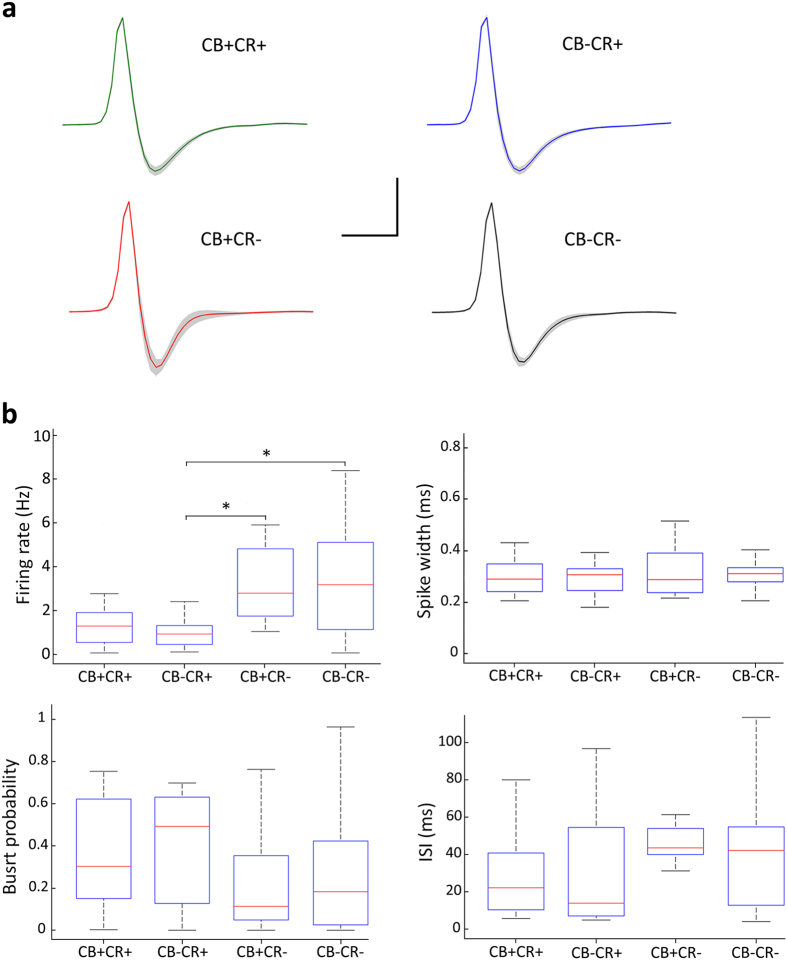
Basic action potential parameters of identified neurons in the midline thalamus. (**a**) Average action potential waveforms from thalamic neuronal populations as defined by their expression of neurochemical markers. Note that spike waveforms are consistently similar between neuronal populations. (**b**) Box plot representations of activity parameters from midline thalamic neurons calculated from the entire recording session. Note differences between CR+ and CR− cells only for spontaneous firing rates. Other parameters were not significantly different. CR+CB+, n = 21 cells; CR+CB−, n = 18 cells; CR−CB+, n = 15 cells; CR−CB−, n = 25 cells. One way ANOVA was used to assess for significant effects between cell populations, and significant differences (asterisks (*), p < 0.05) were then identified post hoc using Tukey’s test (CR+CB− and CR−CB+, p = 0.031; CR+CB− and CR−CB−, p = 0.046). Scale bar: horizontal 1 ms, vertical 0.5 mV.

**Figure 3:**
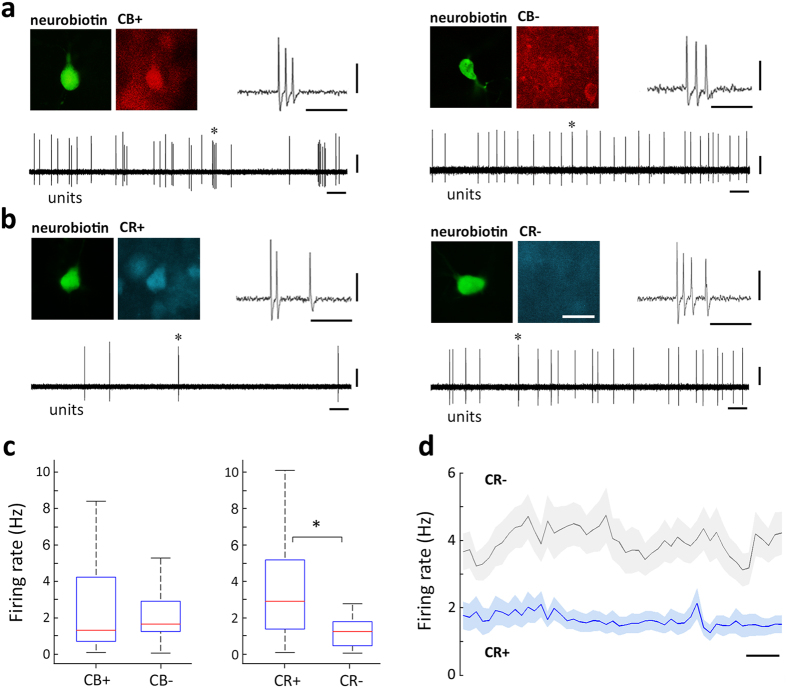
Spontaneous firing patterns of identified neurons in the midline thalamus. (**a**,**b**) Firing patterns and fluorescent micrographs showing immunoreactivity for CB (**a**) or CR (**b**) in four identified midline thalamic neurons, recorded in Re (AL24c1, left) and PC (AL40c2, right) for (**a**) and PV (ED22c7, left) and CM (AL58c5, right) for (**b**). Immunoreactivity for CB and CR is not shown in all cases for clarity; yet AL24c1 and AL40c2 were CR−; whereas AL58c5 and ED22c7 were CB+ (Supplementary Figure 6). Asterisks (*) depict example spike burst, expanded in the inset. (**c**) Box plot representations of the population data show no statistically significant difference in the spontaneous overall mean firing rates of cells when they were separated on the basis of the expression of CB (Mann-Whitney U test, p = 0.745; CB+, n = 36 cells; CB−, n = 43 cells); however, there was a statistically significant difference in the spontaneous mean firing rates when cells from the same pool were sorted according to the expression of CR (Mann-Whitney U test, p = 8.94 × 10^−5^, CR+, n = 39 cells, CR−, n = 40 cells). (**d**) Time resolved spontaneous discharge of thalamic neurons recorded in different sessions and animals sorted by their expression of CR. Time zero marks the onset of recording session for each neuron. Note consistent difference in firing rates between CR+ and CR− cells over time. Average ± SEM. CR+, n = 37 cells; CR−, n = 39 cells. Binsize, 10 s. Scale bars: (**a**) micrographs 25 μm; burst insets, horizontal 25 ms, vertical 0.5 mV; units, horizontal 1 s, vertical 0.5 mV; (**d**) 50 s.

We next quantitatively compared basic physiological parameters of the recorded neurons *in vivo*. Action potential waveforms were similar among the diverse neurochemically defined cell populations (Fig. 2a). We also compared the firing patterns based on the inter-spike intervals that neurons discharged, and found no apparent differences. Thalamic neurons operate in complementary functionally distinct firing modes; namely, tonic or bursting^41^,^42^. Consequently, we sought to compare bursting discharge modes between the neurochemically defined cell populations, and found that the proportion of action potentials discharged in bursts (i.e.; burst probability) was not significantly different between the neuronal populations defined by neurochemical profile, during the entire recording session. We then evaluated neuronal activity in the midline thalamus by comparing the mean firing rate between neuronal populations during the entire recording session. Thus, we observed a difference in spontaneous activity levels. Indeed, on average, CR+ neurons had significantly lower firing rates than CR− cells (Fig. 2b). Given the fact that the expression profile of CB was seemingly uncorrelated with differences in baseline activity levels of midline thalamic neurons, we decided to perform further analysis focusing on the expression profiles of a single calcium-binding protein at a time (i.e.; CR or CB). Consequently, by sorting cells based solely on their expression of CB, we could define two populations with similar number of neurons (Table 2). In that case, we detected no significant differences in the mean firing rate of cells for both groups in CB+ and CB− neurons (Fig. 3a,c). In contrast, by reanalyzing the same neuronal pool, we confirmed a significantly lower mean firing rate of CR+ neurons when compared with CR− cells (Fig. 3b,c). The differences in firing rate were consistent over time, as they could be detected for the entire duration of physiological recordings (Fig. 3d). Importantly, differences detected in the population were cot found locally in the different midline thalamic nuclei, possibly due to the sample size (Supplementary Figure 3). We then compared the structure of burst discharges in midline thalamic neurons (Table 2). We found that CR+ neurons exhibited an overall higher proportion of burst discharges than CR− neurons (Table 2). This is consistent with the tendency seen in Fig. 2b for burst probability. Hence, our results suggest that there are consistent physiological differences associated to the expression profile of CR in midline thalamic neurons, with CR+ cells being less active, but more “bursty”, than CR− cells.

### Calretinin-expressing neurons are not robustly recruited during hippocampal theta oscillations

**Figure 4:**
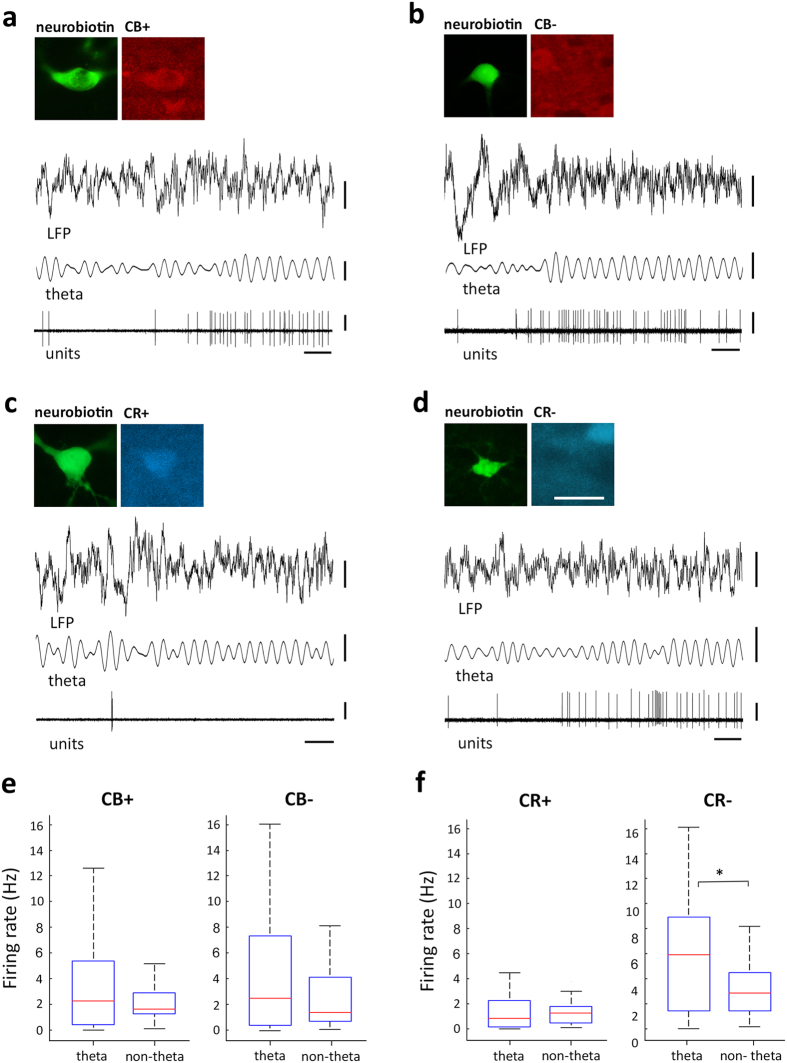
Spike timing of identified neurons in the midline thalamus during transitions to hippocampal theta oscillations. Firing patterns of a midline thalamic CB+ cell (**a**) and a CB− cell (**b**) recorded in VM (AL40c1) and in Sub (AL26c2), respectively. Single cell activity (unit, 0.3–5 kHz) is shown for the transition from non-theta epochs to theta oscillations recorded in the dorsal CA1 area (LFP, 0.3–300 Hz). Theta oscillations are evidenced by LFP filtering (theta, 4–8 Hz) and were automatically detected (see Methods). Note both cells largely increasing their activity during theta oscillations. Immunoreactivity for CR is not shown for clarity; yet AL40c1 and AL26c2 were CR− (Supplementary Figure 6). Firing patterns of a CR+ cell (**c**) and a CR− cell (**d**) recorded in Rh (AL27c3) and Re (AL23c3), respectively. Immunoreactivity for CB is not shown for clarity; yet AL27c3 was CB+ and AL23c3 was CB− (Supplementary Figure 6). Box plot representations of mean firing rates for midline thalamic neurons separated according to the expression of CB (**e**) or CR (**f**). Note that differences in firing rates between non-theta epochs and theta oscillations were significant (asterisk (*)) only for CR−cells. (Wilcoxon signed-rank test, p = 8.64 × 10^−4^). CB+, n = 28 cells; CB−, n = 32 cells; CR−, n = 29 cells; CR−, n = 31 cells. Scale bars: micrographs 25 μm; recordings, horizontal 500 ms, vertical 0.5 mV.

To obtain further insight in and provide context to the different levels of activity in midline thalamic neurons, we next focused on distinct brain states and their associated cortical network oscillations (Table 3). To study correlated patterns of activity between the hippocampus and thalamus, we recorded simultaneously the LFP from the dorsal CA1 and single cell activity from the midline thalamus. Most hippocampal recordings in our experimental conditions (75.9%, n = 60; Supplementary Table 2) presented non-theta periods with spontaneous transitions to theta oscillations (Fig. 4). During hippocampal theta oscillations, nearly half of midline thalamic neurons (53.3%, n = 32) increased their firing rate (121 ± 128% increase, range: 12–694%). Accordingly, we examined if the expression of CR or CB was related to changes in the spike timing of thalamic cells during theta oscillations. There were examples of individual cells dramatically increasing their activity during theta oscillations, and those neurons could be either CB+ (Fig. 4a) or CB− (Fig. 4b). As a population, both CB+ and CB− cells seemed to have the tendency to discharge more during theta oscillations. However, that trend was not statistically significant (Fig. 4e). Hence, the expression of CB held no apparent relation with the spike timing of midline thalamic cells during theta oscillations. Interestingly, when the same neuronal pool was sorted based on the expression of CR, a clear pattern emerged. Indeed, only the activity of CR− neurons was significantly modulated by hippocampal theta oscillations, as transitions between network states were associated with changes in their discharge rate (Fig. 4d,f). Conversely, CR+ neurons did not change their firing rate during theta oscillations (Fig. 4c,e). Furthermore, the firing rates of CR+ and CR− were significantly different during both theta (p = 2.57 × 10^−4^) and non-theta (p = 1.19 × 10^−4^) oscillations, with CR− cells being more active in both network states (Fig. 4f), consistent with their overall higher activity levels described above (Fig. 3). The apparent absence of brain state dependency in the activity levels of CR+ neurons (i.e., during theta and non-theta epochs) could be due to differences in the global network states during which the different neuronal populations (i.e., CR+ vs. CR− cells) were recorded. In order to assess this possibility, we compared basic parameters (i.e., frequency and power) of the brain states associated to the different neuronal populations that we characterized. Our analysis did not reveal any significant difference between brain states, suggesting that differences in neuronal activity were likely related to properties intrinsic to the neuronal populations, rather than global differences in network states (Table 3).

**Figure 5:**
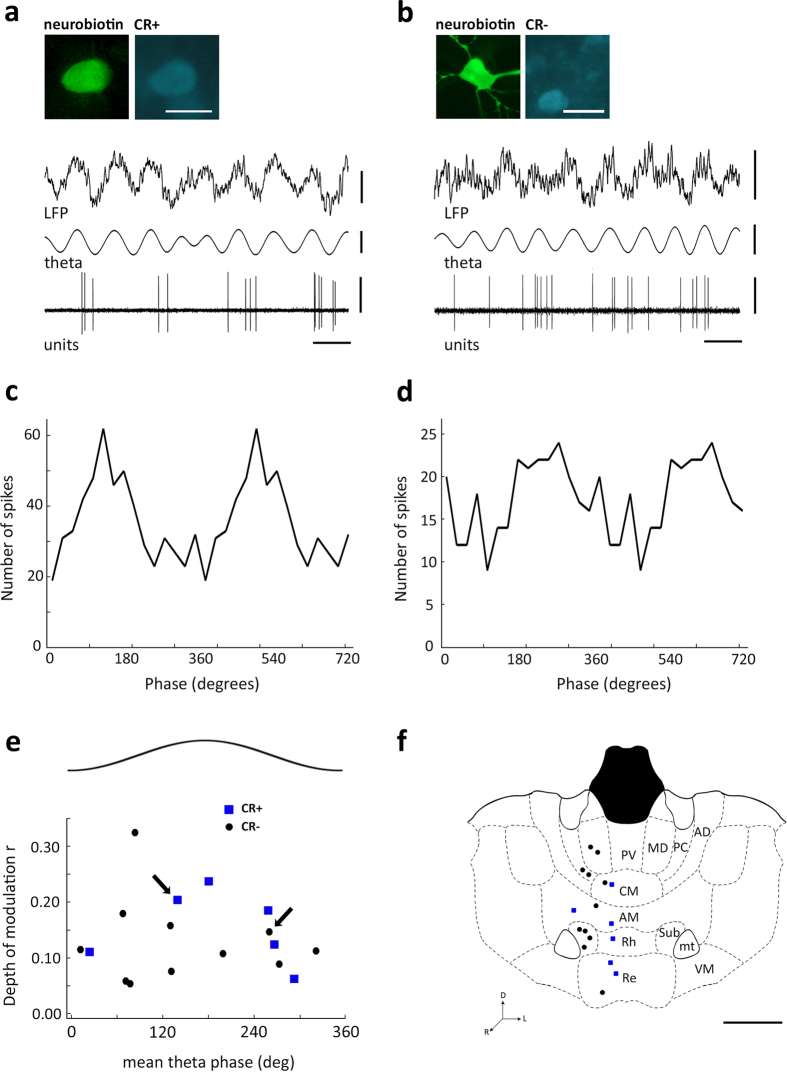
Phase modulation of midline thalamic neurons during hippocampal theta oscillations. (**a**,**b**) Examples of spike timing from a midline thalamic CR+ cell (**a**) and a CR− cell (**b**) in Re (ED15c3) and PC (RC14c2), respectively; recorded during theta oscillations in the dorsal CA1 area (LFP, 0.3–300 Hz). Theta oscillations are evidenced by LFP filtering (theta, 4–8 Hz). Immunoreactivity for CB is not shown for clarity; yet both ED15c3 and RC14c2 were CB+ (Supplementary Figure 6). (**c**,**d**) Examples of theta phase firing probability histograms for the cells depicted in (**a,b**) respectively. The same data are repeated in two cycles for theta histograms to indicate oscillations. The trough of the extracellularly recorded oscillations in dorsal CA1 stratum pyramidale are at 0°, 360° and 720°; bin size: 20°. (**e**) Theta-modulated firing of midline thalamic neurons characterized by the depth of modulation (r) and the mean preferred theta phase angle for each identified cell significantly modulated (Rayleigh test, p < 0.05). The trough and peak of the field theta cycle are at 0° and 180°, respectively. Blue squares, CR+ (n = 6 cells); black circles, CR− (n = 11 cells). Arrows depict data for example histograms from (**b**,**c**). Histograms of average theta phase discharge probability for neurons (Supplementary Figure 5). (**f**) Coronal view of anatomical location of theta-modulated neurons collapsed in one medial plane based of the mouse brain atlas (plate 39^39^). Color code as in (**e**). Scale bars: (**a**) micrographs 25 μm, LFP 0.5 mV, theta 0.2 mV, units 1 mV, horizontal 250 ms; (**f**) 1 mm.

Since many thalamic neurons were active during hippocampal theta waves, we sought to establish if thalamic cells exhibited any phase preference to the oscillation. Even though CR− cells significantly increased their activity during theta episodes, only a fraction of them was phase coupled to the oscillatory cycles (Fig. 5). Indeed, the spike timing of one third of CR− neurons (35.5%, n = 11) was significantly phase-modulated during theta oscillations (Rayleigh test, p < 0.05). To quantify the depth of modulation, we computed a normalized vector sum of the theta phase angles of all spike times for each cell, where the vector length and angle represent depth of modulation and preferred phase, respectively. Our analysis revealed that, on average, CR− cells were weakly modulated and did not exhibit a marked phase preference during theta oscillations (r = 0.13 ± 0.08, phase = 68 ± 66.3°, n = 11). In fact, the discharge of a significantly smaller fraction (binomial test, p = 0.026) of CR+ neurons (21%, n =  6) was also modestly modulated during theta oscillations (Rayleigh test, p < 0.05), with similar characteristics to CR− cells (r = 0.15 ± 0.07, phase = 81.1 ± 70.6°, n = 6). Theta-modulated cells did not exhibit any particular anatomical location (Supplementary Figure 4). Finally, we examined the neuroanatomical location of the theta-modulated neurons and established that they were widely distributed along the dorsoventral axis of the midline thalamus, and thus, were not associated to any specific nucleus or region (Fig. 5f). Thus, our results suggest that CR− midline thalamic neurons are actively engaged during hippocampal theta waves, whereas CR+ neurons, with lower baseline activity levels, are not strongly recruited by the oscillatory episodes. Overall, the spike timing of a sizable fraction of midline thalamic neurons (28.3%, n = 17) was significantly modulated by hippocampal theta oscillations, yet neither the strength of modulation or phase preference were robust (Fig. 5e).

### Calretinin-expressing neurons are inhibited during hippocampal sharp wave-ripples

**Figure 6:**
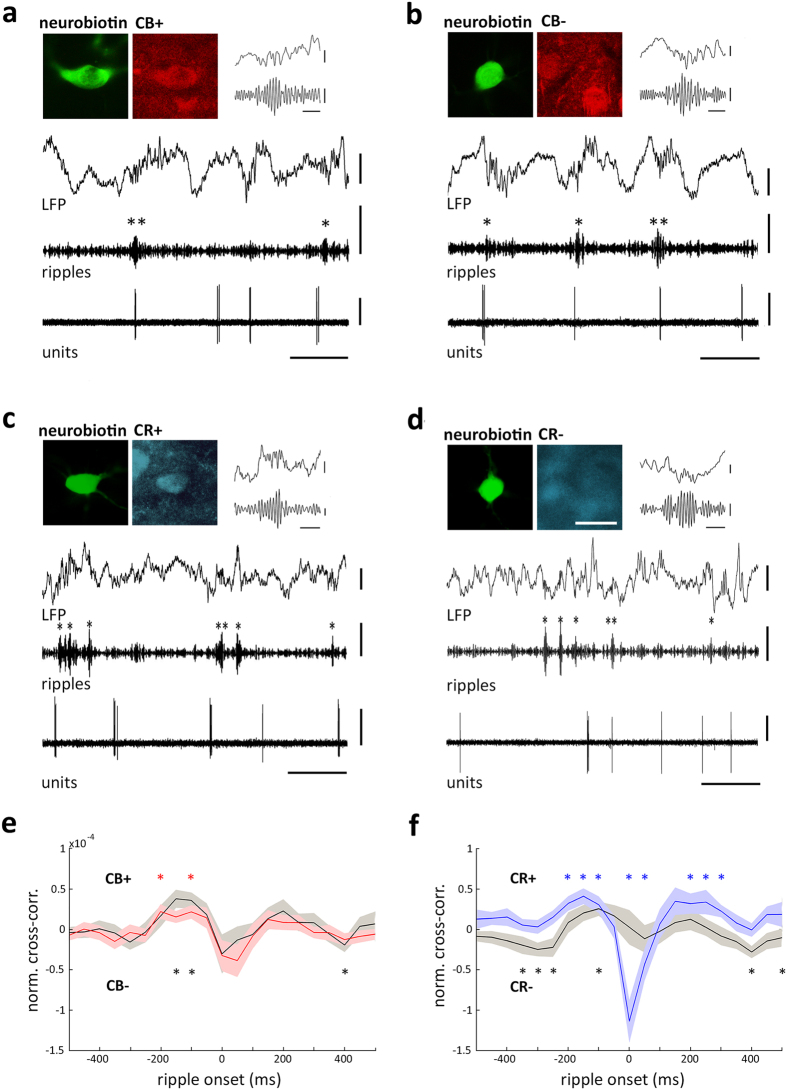
Spike timing of identified neurons in the midline thalamus during hippocampal ripple oscillations. (**a**,**b**) Firing patterns of a midline thalamic CB+ cell (**a**) and a CB− cell (**b**) recorded in VM (AL40c1) and in AM (AL43c1), respectively. Single cell activity (units, 0.3–5 kHz) is shown during non-theta epochs in the dorsal CA1 area (LFP, 0.3–300 Hz). Sharp wave-ripples are evidenced by LFP filtering (ripples, 100–200 Hz). Sharp wave-ripples were detected by filtering and thresholding (see Methods). Single asterisk (*) depict ripple episodes and double asterisks (**) show ripple episodes expanded in insets. Note both cells occasionally discharged in coincidence with hippocampal ripples. Immunoreactivity for CR is not shown for clarity; yet AL40c1 and AL43c1 were CR− (Supplementary Figure 6). (**c**,**d**) Firing patterns of a CR+ cell (**c**) and a CR− cell (**d**) recorded in CM (ED16c5) and Re (RC17c2), respectively. Immunoreactivity for CB is not shown for clarity; yet ED16c5 and RC17c2 were CB+ (Supplementary Figure 6). (**e**,**f**) Normalized cross-correlation (norm. cc) functions between ripples onset and the spike timing of neurons sorted by the expression of CB (**e**) or CR (**f**). Note that neurons when separated into CB+ and CB− populations exhibited similar dynamics during ripple episodes; whereas when using the expression profile of CR, CR+ cells were significantly modulated during ripple episodes. Asterisks (*) depict individual time-points with statistically significant differences from shuffling (Wilcoxon signed-rank test, p < 0.05). Shufflings were controlled for spurious correlations (see Methods). Light areas illustrate standard error for each distribution. In (**e**), Gray and black lines depict average cross-correlograms for CB+ (n = 28 cells) and CB− (n = 37 cells), respectively; whereas in (**f**), Gray and black lines depict average cross-correlograms for CR+ (n = 27 cells) and CR− (n = 38 cells), respectively. Binsize, 50 ms. Scale bars: micrographs 25 μm; LFP 0.5 mV; ripples 0.2 mV; units 0.5 mV; horizontal 500 ms; inset ripples, LFP 0.25 mV, ripples 0.05 mV, horizontal 50 ms.

In our recordings, hippocampal ripples were reflected as short periods of high-frequency activity, taking place exclusively during non-theta epochs. In order to assess the spike timing of individual thalamic neurons with respect to ripple oscillations, we computed the normalized cross-correlation function between simultaneously recorded hippocampal ripples and the action potentials of individual neurons in the midline thalamus, considering the onset of ripple episodes as time reference (Fig. 6). All midline thalamic neurons seemed to slightly increase their discharge probability just before the onset of sharp wave-ripples (Fig. 6a–c). We then reanalyzed the same neuronal pool, but distinguished neurons based on their expression of CR. Hence, we found that CR− neurons did not exhibit major changes in their discharge probability during sharp wave-ripples. In contrast, the spike timing of CR+ neurons was significantly modulated by sharp wave-ripples (Fig. 6d–f). Indeed, the firing rate of CR+ cells was significantly inhibited during ripple episodes; and slightly activated immediately before and after the high frequency episodes (Fig. 6f). Importantly, the temporal correlation between thalamic discharge and hippocampal ripples was not the result of co-modulation by slow oscillatory activity characteristic of non-theta epochs^43^,^44^, as spurious contributions were controlled by shuffling and subtracting surrogate data (see Methods). Hence, these results show that the discharge probability of CR+ neurons in the midline thalamus is selectively modulated during sharp wave-ripples.

## Discussion

By recording and labeling individual neurons in anesthetized mice, we show here evidence for functionally distinct neuronal populations in the midline thalamus. Indeed, our anatomical and physiological characterization leads us to propose that at least two different populations of midline thalamic projection cell deliver differential synaptic output to cortical targets according to ongoing brain states as defined by hippocampal activity. Our data show that rather than anatomical location, a major defining feature in the physiological properties of midline thalamic neurons is the expression of CR, a calcium-binding protein. Hence, distinct thalamic neuronal populations, identified by the somatic expression of CR, are differentially regulated by hippocampal pathways during network rhythms associated to behavioural contexts, likely supporting different stages of memory processing.

Midline thalamic nuclei give rise to large glutamatergic axon terminals that densely distribute within frontal and temporal cortical lobes^27^,^45^. It has been reported that a dense fiber plexus located in the entorhinal cortex contains both CR and vesicular glutamate transporter 2 in the same axons. Complementary retrograde tracing experiments found labeled cell bodies in the nucleus reuniens co-expressing CR^46^. Our results show that CR+ cells are widely expressed across the midline thalamus, and given their anatomical and physiological features suggest that they are likely to be glutamatergic projection cells^1^. Cortical projections from the midline thalamus are not homogeneous across nuclei. Indeed, neurons in dorsal structures preferentially target the medial prefrontal cortex, with little input to the medial temporal lobe^5^,^24^; neurons in middle regions, project almost exclusively to the medial prefrontal cortex^24^,^47^; whereas neurons in ventral areas, project mostly to the parahippocampal cortex, with less significant projections to the medial prefrontal cortex^24^,^27^. Thus, the anatomical organization of synaptic output from the midline thalamus is not homogeneous, with every nucleus displaying distinct efferent connectivity patterns. Interestingly, our results suggest that a major feature in defining the activity pattern of thalamic neurons is not their nuclear location, but the expression profile of calcium-binding proteins, particularly CR.

The different neuronal activity patterns that we describe could be related to the expression of dissimilar intrinsic membrane properties in the midline thalamus. Indeed, we found lower overall spontaneous firing rates and higher proportions of burst discharges in CR+ neurons compared to CR− cells. Both features would be consistent, for example, with more hyperpolarized baseline membrane potential or higher input resistance in CR+ neurons^42^. Further experiments will have to test this possibility with intracellular recordings *in vivo*. Compared to parvalbumin, CR and CB are considered slow Ca^2+^ buffers^10^. Our results suggest that neither of these calcium-binding proteins influences the shape of the action potential, since spike waveforms were similar across thalamic neurons, regardless of their CR or CB expression profile. Nonetheless, both the firing rate and burst incidence of thalamic neurons were correlated with the expression of CR. This does not mean that CR directly affects the biophysical process of spiking discharge, but it reveals a neurochemical correlate of physiological activity which might be useful in the future to define thalamic cell types. Indeed, the expression of CB and CR has proved to be useful to define cell types in other brains structures such as neocortex^14^, hippocampus^13^, striatum^48^ and cerebellum^49^.

The diverse activity patterns detected in midline thalamic neuronal populations could be the result, at least in part, of differential dynamics in synaptic input provided by anatomical innervation arrangements from the hippocampal projection system. Indeed, the subicular formation represents the main output station of the hippocampus and projects extensively to the midline thalamus via the dorsal fornix, reaching bilaterally the reuniens, anteromedial, paraventricular and submedius nuclei^29^,^30^,^31^. Previous studies have found that anterior thalamic nuclei are modulated by hippocampal theta rhythms, likely via subicular projections^25^,^50^,^51^. The ultrastructural organization of the subicular input to the midline thalamus has not been documented in detail. Nevertheless, based on our physiological data, it could be reasonable to speculate that a large proportion of subicular axons terminate directly onto CR− neurons, exciting them during hippocampal theta oscillations. Another plausible option for the relay of theta oscillations is the input provided by the entorhinal cortex, which also projects extensively to most of the midline thalamic nuclei^52^ and expresses robust theta oscillations that are coherent with hippocampal activity^53^. Additional theta-modulated synaptic drive may arise from the medial prefrontal cortex. Based on connectivity patterns, this could seem as a natural alternative, given the prominent innervation provided by the medial prefrontal cortex to the midline thalamus^54^,^55^. Nonetheless, the origin of theta oscillations in the medial prefrontal cortex is controversial, as the rhythm is not concurrently expressed in the hippocampus and medial prefrontal cortex^34^,^56^ and there is evidence that theta waves in prefrontal cortex are the result of volume conduction^57^. Conversely, other studies have suggested that theta waves are synaptically generated in the prefrontal cortex and can synchronize with hippocampal rhythms^58^,^59^,^60^. Certainly, the previous possibilities are not exclusive, and a combination of them could operate concurrently in delivering theta oscillatory information to the midline thalamus during active behavior. Indeed, during theta oscillations, the entorhinal performant path strongly drives hippocampal activity^61^,^62^, thus likely contributing to the theta-modulated subicular output to the midline thalamus. Overall, our results show that the theta rhythm can effectively reach the midline thalamus as it is capable of modulating both the discharge rate and phase-coupling of neuronal activity. Both of these elements might contribute to propagate memory signals and synchronize cortical targets during spatial exploration^33^,^36^.

On the other hand, the hippocampal theta rhythm impinging on the midline thalamus is likely to be supplemented by increased cholinergic tone arising from dense pedunculopontine^63^ and laterodorsal tegmental^64^ projections, which are known to robustly stimulate the thalamus during activated states^65^. An additional mechanism to explain the differential recruitment of thalamic neurons during hippocampal theta oscillations could be related to different expression profiles of muscarinic and nicotinic receptors associated to the expression of CR. Indeed, cholinergic receptors are abundantly expressed in the thalamus^66^,^67^; yet, it is currently unknown if they are segregated in different neuronal populations. Differential expression of cholinergic receptors would be consistent with *in vitro* and *in vivo* data showing wide ranging effects of acetylcholine in thalamic neurons that were not anatomically identified^68^,^69^,^70^,^71^. On other hand, compared to anesthesia preparations the number of theta-modulated neurons is likely to be different in the awake state as the systemic level of neuromodulators is much higher and the network will be actively engaged in faster rhythms. Moreover, different thalamic nuclei show distinct synaptic connectivity, and are accordingly expected to be active during different behavioural contexts^5^. Interestingly, studies in identified neurons in the basal forebrain^72^, hippocampus^73^, and barrel cortex^74^,^75^ have shown that the spike timing is preserved in relation to network oscillations in anesthesia and wakefulness. This is remarkable given that the frequency of network oscillations is faster and the average neuronal firing rates are much higher in awake animals as compared to anesthetized preparations. This suggests a tight control of network structured activity patterns across a wide dynamic range. Thus, we expect that similar results should hold for the thalamus.

Sharp wave-ripple episodes represent another hallmark hippocampal activity pattern, that is prominent during non-theta states^76^,^77^ and associated to episodic memory consolidation^78^,^79^,^80^,^81^. Ripples are locally generated in the hippocampus, but robustly propagate to the cortex^37^,^82^ and produce global effects^37^,^83^, thus defining a brain state. Our data also shows that sharp wave-ripples exert a differential impact on midline thalamic neurons. Recent experiments used NET-fMRI in monkeys to identify the brain areas that consistently modified their activity in relation to sharp wave-ripples. The study showed that ripples were tightly associated with robust cortical activations that occur concurrently with extensive activity suppression in subcortical territories, including: thalamus, basal ganglia, cerebellum, and midbrain–brainstem neuromodulatory structures^83^. Interestingly, our results confirm that the midline thalamus is, at least partially, inhibited during sharp wave-ripples. Moreover, our data suggest that CR+ cells are particularly modulated during sharp wave-ripples as their activity is selectively inhibited. Interestingly, right after the end of ripples, CR+ cells exhibited a small, yet significant, increase in spiking activity, similar to burst rebound responses after active synaptic thalamic inhibition^84^. Since rodents virtually lack GABAergic thalamic interneurons^85^, it reasonable to propose that the zona incerta, the main inhibitory drive of the midline thalamus^86^, might be selectively recruited during hippocampal ripples to control synaptic activity of particular domains, in this case, specific neuronal populations, of the midline thalamus.

Memory formation and decision-making largely depend on the coordinated interaction of the medial temporal lobe and medial prefrontal cortex^87^,^88^. It has been proposed that the medial prefrontal cortex provides context information to the medial temporal lobe during learning and retrieval of position-dependent memories. Similarly, the medial prefrontal cortex could make memory-based inferences by retrieving memories from the medial temporal lobe^89^. We propose that coupling between these multiple oscillatory systems may be a mechanism exploited by the thalamus for linking the medial prefrontal cortex, dedicated to planning and execution, with the medial temporal lobe, dedicated to memory formation and context representation. According to our data the midline thalamus might not be directly associated with the process of memory consolidation, which takes place during sharp wave-ripples, a result consistent with previous data^83^. On the contrary, midline thalamic activity might be detrimental to the process as it seems to be actively inhibited. During theta oscillations, when memory encoding occurs, the activation of a particular thalamic cell population seems to be required to increase cortical excitatory drive. Although at present we do not know the functional consequences of the selective activity patterns displayed by CR+ and CR− cells in the midline thalamus, it is important to remark that they could provide the basis for a differential thalamic contribution to particular stages of memory processing, with CR− neurons being activated by theta oscillations during memory encoding, whereas CR+ neurons are actively inhibited by sharp wave-ripples during memory consolidation. These observations provide support to the presence of specialized neuronal classes in the thalamus, thus sustaining differential synaptic flow between prefrontal medial and temporal lobes^45^,^90^, necessary for memory specificity and generalization^15^,^17^.

## Methods

### *In vivo* recording and labeling

Adequate measures were taken to minimize pain or discomfort in experimental animals. Experiments were carried out in accordance with the guidelines published in the NIH *Guide for the Care and Use of Laboratory Animals* (NIH publication no. 86-23, revised 1987), and reviewed and approved by the university (Pontificia Universidad Catolica de Chile) and funding body (Comision Nacional de Investigacion Cientifica y Tecnologica) bioethics committees. Recordings were obtained from adult C57Bl/6 mice (n = 45, from either sex; 20–30 g, 15–20 weeks). Animals were anesthetized with urethane (0.8 g/kg body weight), plus supplemental doses of ketamine and xylazine (20 and 2 mg/kg, respectively) as needed; body temperature was maintained with a heating pad. The head was placed in a stereotaxic frame, the skull was exposed, and a small craniotomy (~1 mm) was made above the hippocampus (anteroposterior, −2.3 mm; mediolateral, +2.3 mm; coordinates from Bregma^39^) to insert a recording electrode at 20° degrees toward the midline. Another hole in the skull was drilled above the midline thalamus (anteroposterior, −0.8 mm; mediolateral, +0.3 mm). Neuronal activity in the midline thalamus was recorded extracellularly with a glass electrode (10–20 MΩ, *in situ* descended 3–5 mm in the dorsoventral axis) filled with 1.5% neurobiotin (Vector Laboratories) in 0.5 M NaCl, and the reference local field potential (LFP) was recorded in the hippocampus with a second glass electrode (10–20 MΩ) located as close as possible to the dorsal CA1 stratum pyramidale, for which the electrode was descended 0.8–1 mm, until ripple oscillations were visually detected online^91^. Single-unit activity and LFP (sampling rate 10 kHz) were analog filtered between 300 Hz–5 kHz and 0.3 Hz–2 kHz, respectively. All of the extracellularly-recorded thalamic cells were individually labeled with neurobiotin using the juxtacellular labeling method, only after data for the firing patterns had been sampled from the unaffected cell. Spike shape and amplitude were monitored during recording and labeling to ensure that the same cell was recorded and labeled. Two to 4 h after labeling, the mice were terminally anesthetized and cardiac perfusion with saline was followed by 20 min fixation with a fixative of 4% paraformaldehyde. Brains were extracted and sectioned coronally (60–70 µm thickness), and sections were further processed for epifluorescence microscopic visualization of labeled neurons. Location of labeled neurons was established in reference to standard brain atlas coordinates^39^.

### Brain-state and time-frequency analysis

We defined brain-states based on the hippocampal LFP. We recognized theta oscillations, non-theta epochs and ripple episodes, in consistency with previous studies^91^. Unless stated, the LFP from dorsal CA1 stratum pyramidale was considered as the time-frame reference for the spike-timing of recorded cells. Theta oscillations were detected by calculating the continuous ratio between the envelopes of theta (4–8 Hz) and delta (2–3 Hz) frequency bands filtered from the hippocampus LFP, and calculated by the Hilbert transform. A ratio of 1.4 SD or higher, during at least 2 s defined epochs of theta oscillations. Recording episodes outside theta oscillations were defined as non-theta epochs. To determine the phase relationship between single-cell activity and theta cycles, the local field potential during theta episodes was filtered between 4 and 8 Hz, and the troughs of the theta oscillations were detected in the filtered signals. Sharp wave-ripples were recorded in dorsal CA1, as close as possible to stratum pyramidale (Fig. 6) and considered as the time-frame reference for the spike-timing of the recorded neurons and population activity (LFP) in the thalamus. We used a recently described method for ripples detection^83^ with some variation. Briefly, the hippocampus LFP was first down-sampled to 1 kHz, then band-pass filtered (100–200 Hz) using a zero phase shift non-causal finite impulse filter with 0.5 Hz roll-off. Next, the signal was rectified and low‐pass filtered at 20 Hz with a 4th order Butterworth filter. This procedure yields a smooth envelope of the filtered signal, which was then z‐score normalized using the mean and SD of the whole signal in the time domain. Epochs during which the normalized signal exceeded a 3.5 SD threshold were considered as ripple events. The first point before threshold that reached 1 SD was considered the onset and the first one after threshold to reach 1 SD as the end of events. The difference between onset and end of events was used to estimate the ripple duration. We introduced a 50 ms-refractory window to prevent double detections. In order to precisely determine the mean frequency, amplitude, and duration of each event, we performed a spectral analysis using Morlet complex wavelets of seven cycles. The Matlab toolbox used is available online as LANtoolbox (http://lantoolbox.wikispaces.com/).

### Cross-correlation analysis

Activity of thalamic neurons and hippocampal ripples was cross-correlated by applying the “sliding-sweeps” algorithm^92^. A time window of ±1 s was defined with the 0 point assigned to the start time of a ripple. The timestamps of the thalamic spikes within the time window were considered as a template and were represented by a vector of spikes relative to t = 0 s, with a time bin of 50 ms and normalized to the total number of spikes. Thus, the central bin of the vector contained the ratio between the number of thalamic spikes elicited between ±25 ms and the total number of spikes within the template. Next, the window was shifted to successive ripples throughout the recording session, and an array of recurrences of templates was obtained.

Both thalamic timestamps and start times of ripples where shuffled by randomized exchange of the original inter-event intervals^93^ and the cross-correlation procedure was performed on the pseudo-random sequence. The statistical significance of the observed repetition of spike sequences was assessed by comparing, bin to bin, the original sequence with the shuffled sequence. An original correlation sequence that presented a statistical distribution different from 100 simulated shufflings was considered as statistically significant, with p < 0.01 probability, instead of a chance occurrence (see *Statistics*). For every recording with a significant correlation, the average of the simulated shuffling was subtracted from the average of the correlation curve and a representative cross-correlogram was obtained. To reveal repeating event correlations through the population, all representative cross-correlogram curves were pooled together and the statistical significance of a non-zero observed value was computed (see *Statistics*).

### Detection of Low-Threshold Spike bursts

Thalamic projection neurons discharge low-threshold Ca2+ spike bursts by of various species during anesthesia, natural sleep, and quiet wakefulness^41^,^94^,^95^,^96^. Previous analyses have shown that spike bursts are preceded by a silent period (50–100 ms^97^). Spike bursts were extracted according to established criteria used in extracellular unit recordings^84^,^98^. Accordingly, at least 2 action potentials with an inter spike interval of ≤5 ms, but with a preceding silent period of >100 ms^97^; and a maximum inter spike interval of 10 ms was used to define the end of spike burst^96^.

### Tissue processing and anatomical analysis

Neurobiotin-labelled cells were revealed by streptavidin conjugated with Alexa Fluor 488. Once located in the corresponding brain section, cells were photographed in other wavelengths to test for bleed through (false positives). After fluorescence testing, horseradish-peroxidase reactions for light microscopy were performed to reveal somatodendritic structure and axonal labelling, with all necessary controls, as described previously^99^. Briefly, for immunocytochemistry, sections were rinsed three times for 10 min each with phosphate buffer (PB), incubated in 1% horse serum supplemented with 0.3% Triton X-100 in PB for 1 h, and then incubated in 1:2000 dilutions of the CR (mouse, code CG1, Swant Inc.) and/or CB (rabbit, code CB-38a, Swant Inc.) antibody for 24 h at 4 °C, followed by a 1:1000 dilution of the secondary antibody for 3–6 hours at room temperature. Secondary antibodies were conjugated to Alexa Fluor 350, 488, 568, or 660 (Invitrogen); and cells were photographed with the appropriate filter cubes (Nikon; UV-2E/C, B-2E-C, G-2E/C, and Cy5-HYQ, respectively) with an epifluorescence microscope (Nikon Eclipse Ci). Antibody dilutions were performed in PB supplemented with 1% horse serum and 0.3% Triton X-100. Sections were mounted on slides with mounting medium and photographed under epiluminescence microscopy. After that, sections were dismounted and rinsed three times for 10 min each in PB, then processed with an ABC kit (Vector Laboratories). Samples were rinsed, and the peroxidase reaction was developed with 0.05% 3,3-diaminobenzidine-4 HCl (DAB) and 0.003% H_2_O_2_. Sections were mounted on gelatin-coated slides, air-dried, dipped in alcohol/xylene battery for development. Sections were then mounted and photographed under bright-field microscopy.

### Statistics

Unless stated, all tabulated data are presented as the mean ± SD and significant differences were accepted at p < 0.05. For cross-correlation analysis, a Wilcoxon test was applied to compare two independent samples (rank-sum test) as well as to identify a distribution with a median equal to zero (signed-rank test). Corrected p-values with the false discovery rate method^100^ were used to examine statistical significances.

Phase modulation of action potentials was determined by Rayleigh circular statistics^101^. For all circular statistical tests the non-uniformity of the phase distribution, due to skewness of the slow oscillation wave shape, was taken into account using the cumulative density function-based transformation^98^,^102^. Group comparison tests of circular variables were performed using circular ANOVA.

## Additional Information

**How to cite this article**: Lara-Vásquez, A. *et al.* Midline thalamic neurons are differentially engaged during hippocampus network oscillations. *Sci. Rep.* **6**, 29807; doi: 10.1038/srep29807 (2016).

## Supplementary Material

Supplementary Information

## Figures and Tables

**Table 1:** Selective expression of the neurochemical markers calbindin (CB) and calretinin (CR) in neurons recorded from midline thalamic nuclei.

Nucleus	CB +CR+ cells	CB−CR+ cells	CB+CR− cells	CB−CR− cells	Total
MD	0	1	2	2	5
PV	8	3	0	0	11
PC	1	1	1	3	6
PT	0	0	0	1	1
CM	2	2	3	1	8
AM	2	4	3	4	13
Rh	2	4	0	2	8
Re	5	3	3	6	17
Sub	0	0	1	6	7
VM	1	0	2	0	3
Total	21	18	15	25	79

Abbreviations: MD, mediodorsal nucleus; PV, paraventricular nucleus; PC, paracentral nucleus; PT, paratenial nucleus; CM, centro medial nucleus; AM, anteromedial nucleus; Rh, rhomboidal nucleus; Re, reuniens nucleus; Sub, submedial nucleus; VM, ventromedial nucleus.

**Table 2:** Discharge parameters of identified neurons in the midline thalamus.

		CB+ cells	CB− cells	P	CR+ cells	CR− cells	P
Firing rate	mean (Hz)	2.63 ± 0.43	2.57 ± 0.35	0.7453	1.64 ± 0.29	3.52 ± 0.41	***0.00009***
	*n*	*36*	*43*		*39*	*40*	
	theta oscillations (Hz)	3.31 ± 0.68	4.49 ± 0.87	0.4722	1.68 ± 0.40	6.05 ± 0.87	***0.00026***
	*n*	*28*	*32*		*29*	*31*	
	non-theta episodes (Hz)	2.56 ± 0.42	2.46 ± 0.33	0.8171	1.67 ± 0.30	3.32 ± 0.38	***0.00012***
	*n*	*36*	*43*		*39*	*40*	
Burst discharge	burst probability	0.29 ± 0.04	0.32 ± 0.04	0.7330	0.39 ± 0.04	0.24 ± 0.04	***0.0122***
	spikes/burst	2.71 ± 0.09	2.61 ± 0.09	0.3578	2.58 ± 0.09	2.72 ± 0.09	0.3357
	burst ISI (ms)	3.93 ± 0.15	3.95 ± 0.16	0.6585	4.15 ± 0.14	3.77 ± 0.16	***0.0466***
	*n*	*33*	*37*		*32*	*38*	

Exact Mann–Whitney *U* test was used for statistical comparisons between populations. Significant differences were accepted at *p* < 0.05. Values are mean ± SEM.

**Table 3:** Characteristics of brain states as defined by network oscillations in the hippocampus.

		CB+ cells	CB− cells	p	CR+ cells	CR− cells	p
Theta oscillations	Frequency (Hz)	5.03 ± 0.06	5.21 ± 0.08	0.1846	5.15 ± 0.09	5.11 ± 0.07	0.8014
	Power (mV/Hz)	7.35E-6 ± 1.13E-6	7.04E-6 ± 1.24E-6	0.5937	7.51E-6 ± 1.34E-6	6.88E-6 ± 1.06E-6	0.7786
	*n*	*28*	*32*		*29*	*31*	
Non-theta epochs	Frequency (Hz)	1.64 ± 0.11	1.69 ± 0.10	0.7941	1.74 ± 0.11	1.60 ± 0.09	0.3076
	Power (mV/Hz)	1.39E-4 ± 2.70E-5	1.41E-4 ± 2.60E-5	0.9372	1.27E-4 ± 2.47E-5	1.53E-4 ± 2.80E-5	0.3465
	*n*	*36*	*43*		*39*	*40*	
Sharp wave-ripples	Frequency (Hz)	102.28 ± 2.62	97.59 ± 2.37	0.2556	100.45 ± 2.66	99.03 ± 2.37	0.7799
	Power (z-score)	3.30 ± 0.18	3.53 ± 0.12	0.5190	3.65 ± 0.16	3.60 ± 0.13	0.9180
	*n*	*36*	*43*		*39*	*40*	

Exact Mann–Whitney *U* test was used for statistical comparisons between populations. Significant differences were accepted at *p* < 0.05. Values are mean ± SEM.
