# Supplementary material for: Midline thalamic neurons are differentially engaged during hippocampus network oscillations
Source: Sci Rep. 2016 Jul 14;6:29807. doi: 10.1038/srep29807 (PMC4944155; doi:10.1038/srep29807)
Supplement: Supplementary Information [file srep29807-s1.pdf]

Midline thalamic neurons are differentially engaged during hippocampus network oscillations

Ariel Lara, Nelson Espinosa, Ernesto Duran, Marcelo Stockle, Pablo Fuentealba

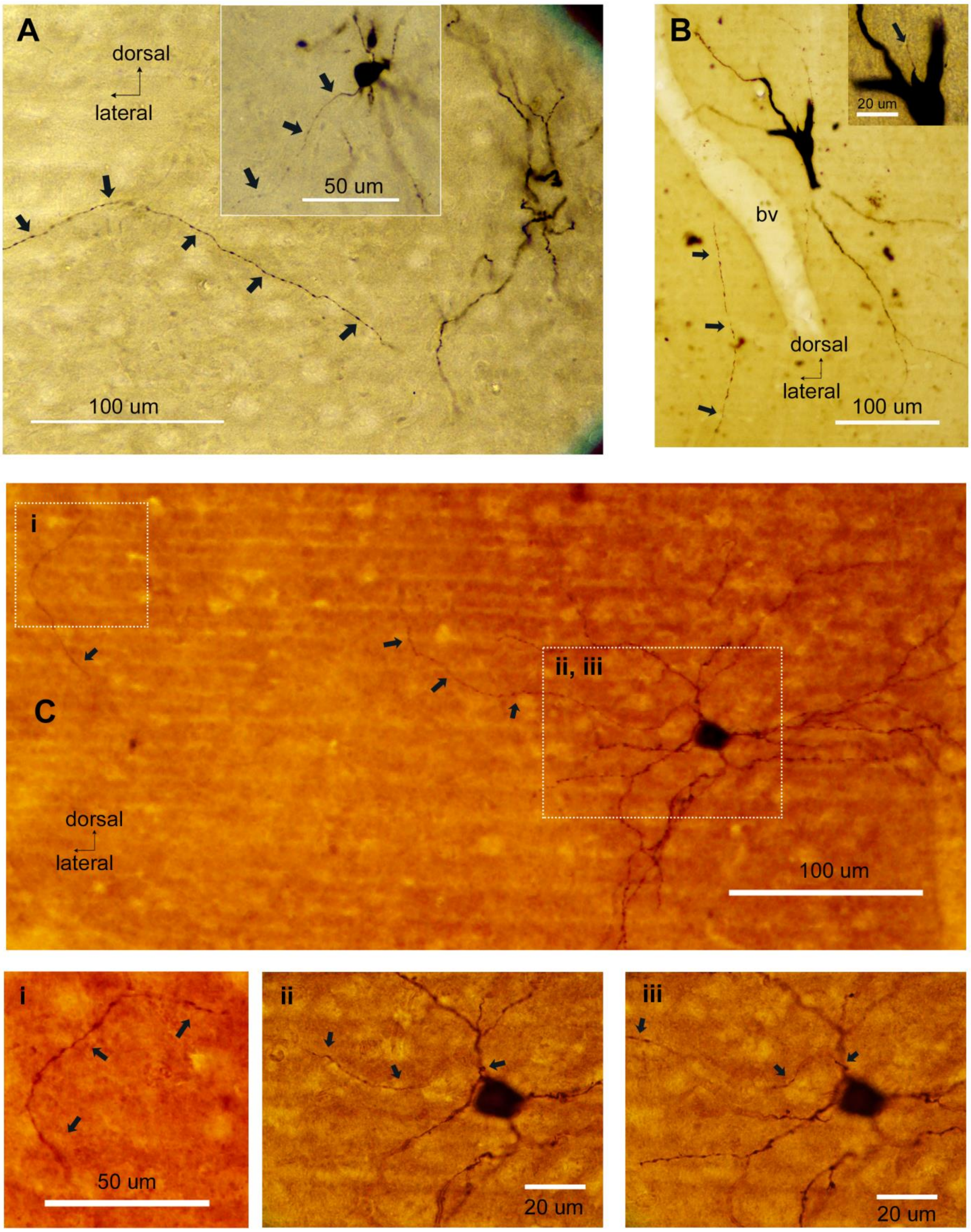

Supplementary Figure 1. Axons from midline thalamic neurons. A, arrows depict main axon from cell AL27c3 projecting away from the midline, with no local collaterals (section 66). Inset shows soma and axon initial segment (section 65). B, arrows depict main axon from cell AL57c4 (section 56) projecting towards the ventral thalamus with no obvious branching. Inset shows axon initial segment arising from the soma in the same section. bv, blood vessel. C, arrows show main axon from cell ED46c3 (section 54) projecting to lateral regions. Dashed rectangles depict a segment of the main

axon away from the soma (i) and the axon initial segment emerging from the soma (ii, iii) at two slightly different focal planes.

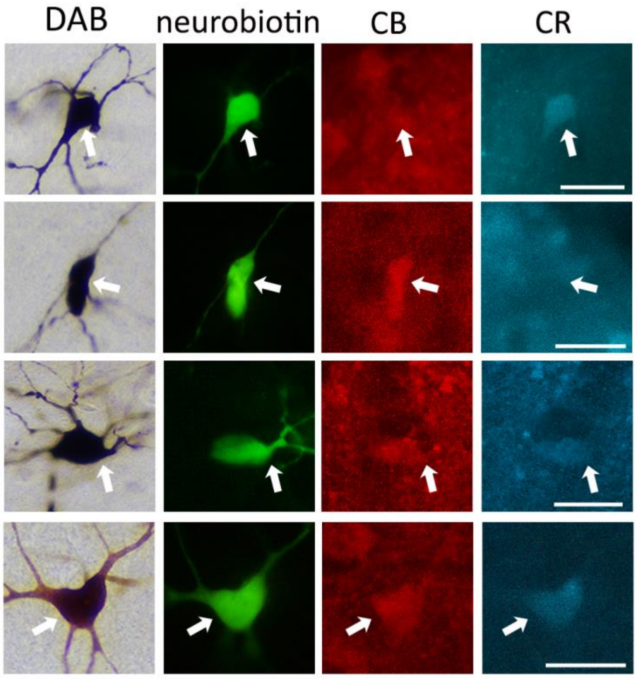

Supplementary Figure 2. Neurochemical profiles of DAB horseradish peroxidase product-labeled thalamic cells from Figure 2. Top panels, cell AL53c7; second middle panels, AL35c2, third middle panels, AL19c1; bottom panels, ED01c3. Scale bars: 25 µm.

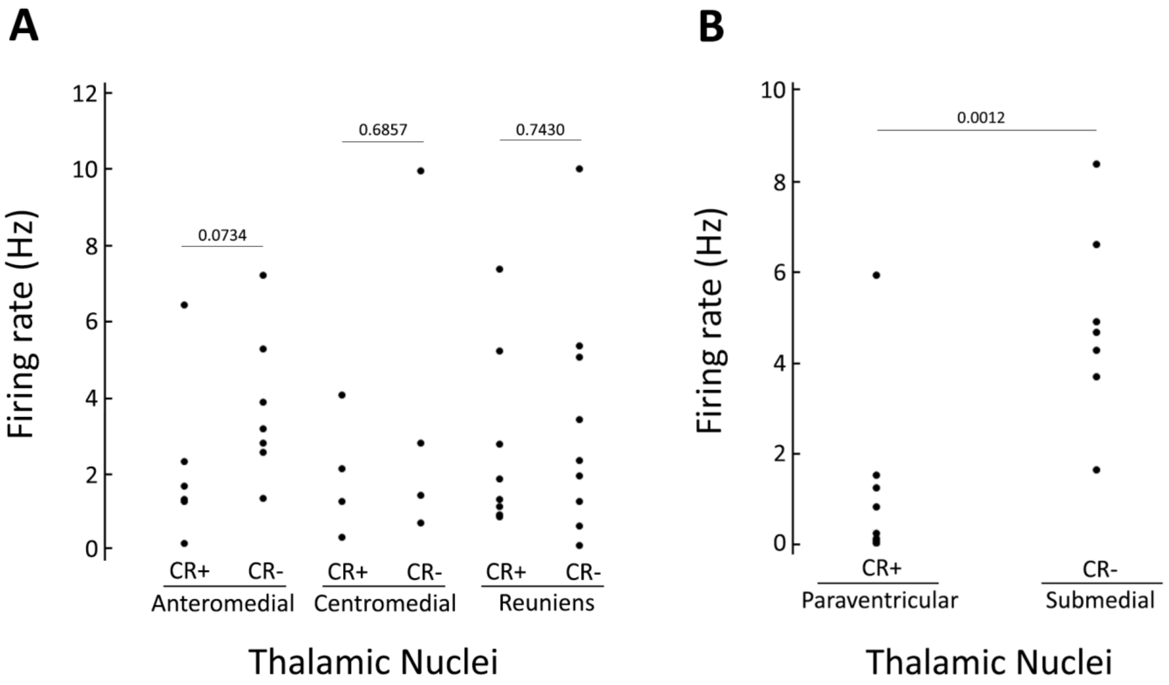

Supplementary Figure 3. Overall firing rates of identified midline thalamic neurons. A, Average firing rates of individual neurons sorted by neurochemical profile (i.e., CR expression) and anatomical location (i.e., midline nucleus). Only groups containing at least 4 cells per category were analyzed. Accordingly, 3 nuclei could be considered for this section. Note that there was no significant difference between CR+ and CR- cells in this analysis. However, the highest firing rates were always exhibited by CR- cells, and when combining all cells together, CR- cells showed significantly higher firing rates (Figure 4). B, Average firing rates of individual neurons in 2 nuclei that contained either only CR+ cells (paraventricular) or only CR- cells (submedial). Note statistical significant difference in this case.

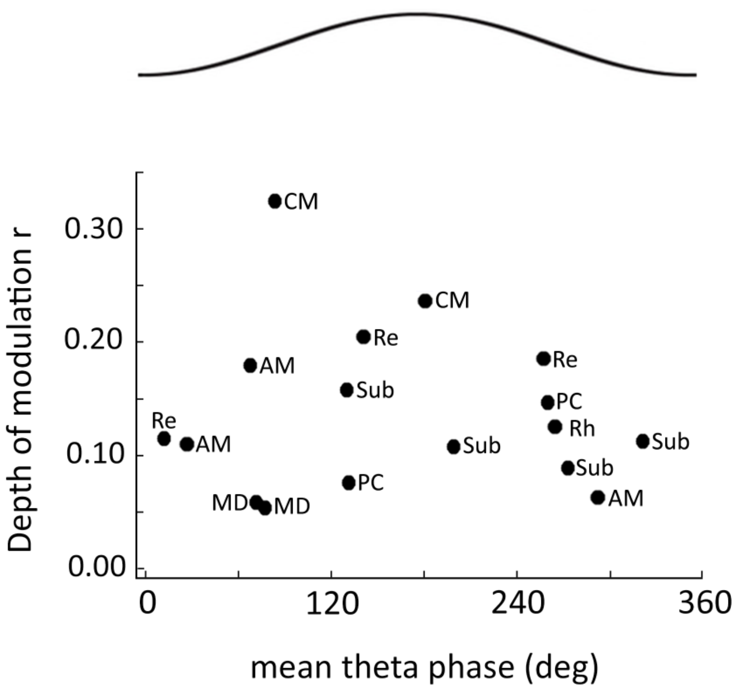

Supplementary Figure 4. Phase modulation of midline thalamic neurons during hippocampal theta oscillations. Theta-modulated firing of midline thalamic neurons characterized by the depth of modulation (r) and the mean preferred theta phase angle for each identified cell significantly modulated (Rayleigh test, $p < 0.05$). The trough and peak of the field theta cycle are at 0° and 180°, respectively. AM, anteromedial nucleus; CM, centromedial nucleus; MD, mediodorsal nucleus; PC, paracentral nucleus; Re, reuniens nucleus; Rh, rhomboidal nucleus; Sub, submedial nucleus.

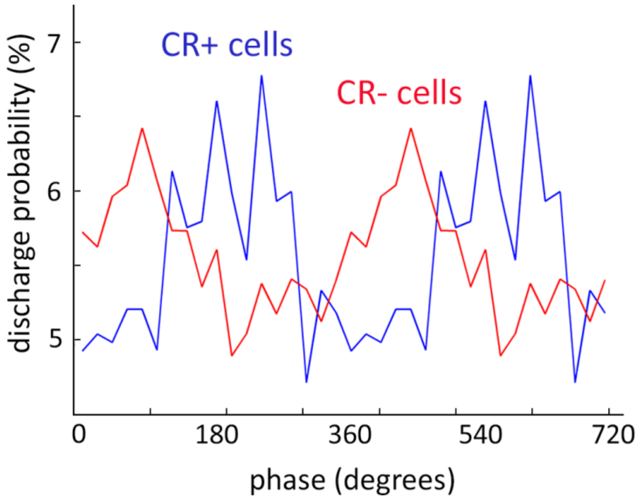

Supplementary Figure 5. Histograms of average theta phase discharge probability for the cells depicted in Figure 5. Number of cells was small (CR+, n = 6; CR-, n = 11); consequently the Watson–Williams F-test could not be applied to detect differences in mean phase angles of neuronal populations. The same data are repeated in two cycles for theta histograms to indicate oscillations. The trough of the extracellularly recorded oscillations in dorsal CA1 stratum pyramidale are at 0°, 360° and 720°. Bin size: 18°.

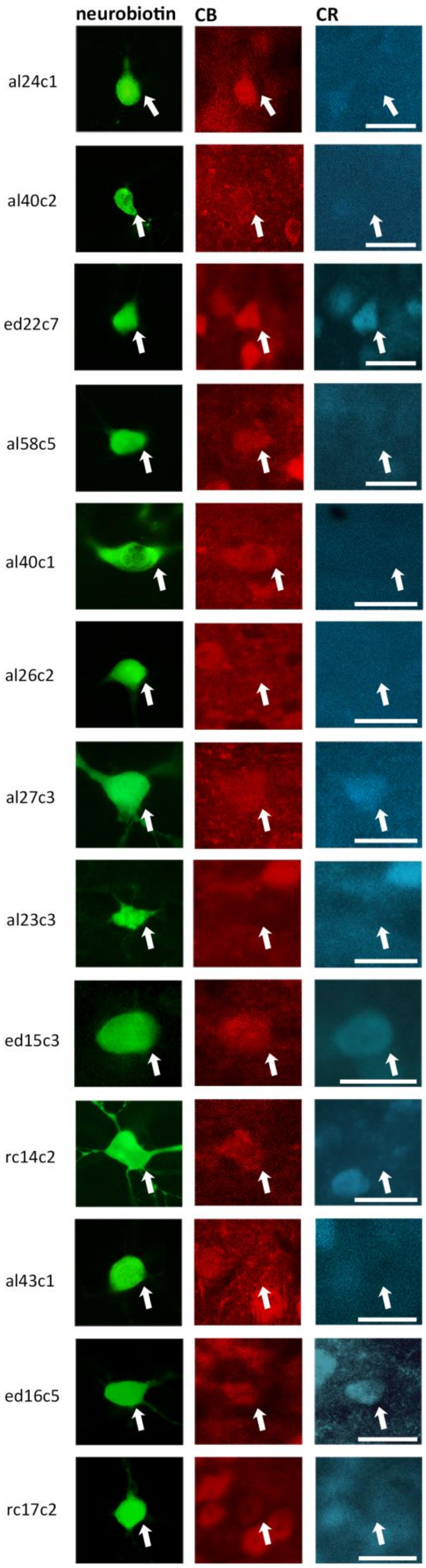

Supplementary Figure 6. Neurochemical profiles of recorded midline thalamic neurons. Expression of calcium-binding proteins from cells presented in Figures 3-6. Scale bars: 25 µm.

ID	CB	CR	nucleus	depth (um)
al12cell2	0	0	AM	3760
al12cell3	0	0	AM	3680
al12cell4	0	0	AM	3620
al13cell1	0	1	CM	3490
al17cell1	0	0	Sub	4020
al17cell2	1	1	PC	3750
al19cell1	1	1	AM	3580
al19cell2	1	1	Re	4150
al20cell1	0	1	Re	4220
al20cell2	1	1	Rh	3700
al20cell4	1	0	Re	3900
al23cell3	0	0	Re	4170
al24cell1	1	0	Re	3890
al24cell3	0	1	Re	4390
al26cell1	1	0	Sub	3950
al26cell2	0	0	Sub	3870
al26cell3	0	0	Sub	3600
al27cell2	1	1	Re	3980
al27cell3	1	1	Rh	3670
al28cell2	0	1	AM	3400
al30cell1	0	0	Re	4120
al34cell2	1	0	AM	3280
al35cell2	1	0	MD	2820
al37cell2	0	0	MD	2830
al40cell1	1	0	VM	3830
al40cell2	0	0	PC	2900
al43cell1	0	0	AM	3910
al44cell1	0	0	Re	3900
al46cell2	1	0	AM	3400
al47cell3	1	1	AM	3550
al47cell4	0	0	CM	3430
al48cell2	0	0	Sub	3600
al51cell1	0	0	Sub	3780
al52cell2	0	1	Rh	3620
al52cell5	0	1	AM	3500
al53cell4	0	1	Re	3830
al53cell7	0	1	AM	3460
al55cell1	0	1	PV	3100
al55cell2	0	0	Re	3830
al55cell3	1	1	PV	3330
al55cell4	0	1	PV	3120
al56cell3	1	1	CM	3350

al56cell4	1	1	PV	2820
al57cell2	0	1	CM	3550
al57cell4	0	1	MD	3160
al58cell3	0	1	PV	2890
al58cell5	1	0	CM	3070
al63cell2	1	1	VM	3780
al63cell3	0	1	PC	3150
al63cell5	0	1	AM	3630
ed01cell3	1	1	Re	3790
ed06cell3	0	0	Re	3790
ed06cell4	1	1	PV	3100
ed08cell4	0	0	MD	2940
ed11cell3	0	0	PC	3330
ed13cell2	1	0	CM	3000
ed14cell2	0	0	PT	3560
ed15cell1	0	1	Rh	3700
ed15cell3	1	1	Re	3880
ed16cell5	1	1	CM	3500
ed17cell1	0	0	Rh	3870
ed18cell4	1	0	MD	3160
ed18cell5	0	1	Rh	3620
ed18cell8	0	1	Rh	3690
ed20cell1	0	0	Rh	3710
ed20cell2	1	1	Re	3780
ed20cell4	1	1	PV	3000
ed20cell5	1	1	PV	3100
ed22cell4	1	1	PV	2980
ed22cell5	1	1	PV	2970
ed22cell7	1	1	PV	3030
rc12cell1	1	0	VM	3800
rc14cell2	1	0	PC	3820
rc17cell2	1	0	Re	4060
rc17cell3	1	0	AM	3450
rc18cell1	0	0	Re	4150
rc18cell2	0	0	PC	3340
rc20cell2	0	0	Sub	3800
rc20cell3	1	0	CM	3320

Supplementary Table 1. List of all anatomically identified neurons (n = 79) with their record identification (ID) and neurochemical profile, based on the expression (1, immunopositive; 0, immunonegative) of calbindin (CB) and calretinin (CR). Examples can be seen in Supplementary Figures 2 and 3. Also listed is the thalamic nucleus were the cell was located, based on the Mouse Brain Atlas [1], and the depth at which cells were recorded, as reported by the micropositioner system. Abbreviations: MD, mediodorsal nucleus; PV, paraventricular nucleus; PC, paracentral nucleus; PT,

paratenial nucleus; CM, centromedial nucleus; AM, anteromedial nucleus; Rh, rhomboidal nucleus; Re, reuniens nucleus; Sub, submedial nucleus; VM, ventromedial nucleus.

Franklin KBJ, Paxinos G (2007) The Mouse Brain in Stereotaxic Coordinates: Academic Press.

ID	nucleus	firing rate (Hz)			burst discharge		
		mean	theta oscillations	non-theta episodes	burst probability	spikes/burst	burst ISI (ms)
al12cell2	AM	7.27	9.62	5.96	0.00	2.50	4.00
al12cell3	AM	3.18	3.72	3.02	0.11	3.00	5.05
al12cell4	AM	2.79	0.32	3.21	0.97	2.92	3.13
al13cell1	CM	4.07	4.47	4.01			
al17cell1	Sub	3.71	15.85	3.62	0.18	2.51	3.20
al17cell2	PC	0.63	3.06	0.44	0.15	2.21	3.46
al19cell1	AM	1.64	3.20	1.50	0.31	3.07	3.41
al19cell2	Re	1.85	2.68	1.84	0.75	3.34	4.33
al20cell1	Re	0.83	0.71	0.83	0.00	2.00	5.00
al20cell2	Rh	1.58	0.29	1.58	0.32	2.65	3.42
al20cell4	Re	10.10		10.10	0.06	3.18	3.30
al23cell3	Re	5.08	14.57	4.85	0.29	3.18	4.27
al24cell1	Re	2.34	1.09	2.43	0.33	2.98	3.48
al24cell3	Re	1.11		1.11			
al26cell1	Sub	1.68	2.34	1.59	0.30	2.46	3.86
al26cell2	Sub	4.70	6.54	4.34	0.31	3.33	3.52
al26cell3	Sub	6.64	16.07	5.55	0.18	2.47	3.89
al27cell2	Re	2.78	2.11	3.01	0.12	2.92	4.05
al27cell3	Rh	1.40	0.83	1.61	0.75	3.11	4.09
al28cell2	AM	6.48	6.04	6.79	0.01	2.10	3.50
al30cell1	Re	5.40	7.73	4.24	0.08	3.14	4.21
al34cell2	AM	2.56	3.79	2.33	0.11	2.66	4.29
al35cell2	MD	5.91	11.46	5.57	0.05	3.58	4.74
al37cell2	MD	6.52	5.15	6.82	0.00	4.00	5.00
al40cell1	VM	3.00	8.29	2.77	0.36	2.96	3.26
al40cell2	PC	1.80	0.30	1.89	0.85	2.73	3.32
al43cell1	AM	3.89		3.89	0.51	2.43	3.88
al44cell1	Re	1.92	3.78	1.91	0.00	2.00	1.00
al46cell2	AM	1.32	0.47	1.33	0.00	2.00	5.00
al47cell3	AM	1.23	1.11	1.23	0.45	3.42	5.33
al47cell4	CM	0.67		0.67			
al48cell2	Sub	4.31	6.32	4.19	0.21	2.88	3.68
al51cell1	Sub	4.92	8.90	4.56	0.45	3.08	3.00
al52cell2	Rh	0.57	0.76	0.49	0.70	3.09	4.00
al52cell5	AM	1.29	0.00	1.30	0.63	3.29	4.65
al53cell4	Re	1.30	1.17	1.38	0.37	3.52	5.06
al53cell7	AM	0.14	0.13	0.18			
al55cell1	PV	0.18	0.28	0.08			

al55cell2	Re	0.59		0.59	0.45	2.14	4.73
al55cell3	PV	0.07	0.00	0.07			
al55cell4	PV	0.12		0.12	0.09	2.00	4.63
al56cell3	CM	1.24		1.24	0.71	2.89	4.99
al56cell4	PV	0.11	0.15	0.11	0.06	2.00	1.00
al57cell2	CM	0.30		0.30	0.49	2.46	4.45
al57cell4	MD	0.46	0.48	0.45	0.13	2.06	4.07
al58cell3	PV	0.87		0.87	0.64	2.10	4.28
al58cell5	CM	1.42	0.22	1.45	0.76	2.80	3.95
al63cell2	VM	1.30	0.55	1.45	0.71	2.50	3.72
al63cell3	PC	0.53	0.11	0.58	0.50	3.00	4.68
al63cell5	AM	2.30	1.17	2.33	0.59	2.43	4.07
ed01cell3	Re	7.45	0.13	8.09	0.31	3.56	3.02
ed06cell3	Re	0.08	0.04	0.11	0.30	2.50	5.00
ed06cell4	PV	1.56	2.06	1.46	0.00	2.00	5.00
ed08cell4	MD	3.16	7.01	2.72	0.01	2.31	3.40
ed11cell3	PC	0.86		0.86	0.01	2.00	4.50
ed13cell2	CM	2.81	2.67	2.85	0.38	3.50	2.69
ed14cell2	PT	0.90	0.00	0.94	0.36	2.24	4.15
ed15cell1	Rh	0.99	1.16	0.99	0.48	2.10	4.12
ed15cell3	Re	5.26	6.10	5.17	0.29	2.80	4.58
ed16cell5	CM	2.12	8.57	2.11	0.62	2.53	3.78
ed17cell1	Rh	1.32		1.32	0.47	2.06	4.10
ed18cell4	MD	1.05		1.05	0.42	2.62	4.64
ed18cell5	Rh	1.32	1.33	1.32	0.69	2.17	4.04
ed18cell8	Rh	2.42		2.42	0.58	2.15	3.98
ed20cell1	Rh	0.45		0.45	0.06	2.00	4.90
ed20cell2	Re	0.90		0.91	0.24	2.57	4.38
ed20cell4	PV	0.28		0.28	0.11	2.00	4.57
ed20cell5	PV	1.28		1.28	0.26	2.69	4.55
ed22cell4	PV	0.09		0.09			
ed22cell5	PV	5.96		5.96			
ed22cell7	PV	0.07		0.07	0.30	2.00	4.43
rc12cell1	VM	2.85		2.85	0.00	2.00	3.00
rc14cell2	PC	2.01	8.12	1.84	0.09	2.52	3.75
rc17cell2	Re	3.44	4.89	2.96	0.14	2.40	3.68
rc17cell3	AM	5.30	5.88	4.20	0.02	2.18	3.59
rc18cell1	Re	1.23	0.71	1.39	0.00	3.00	0.50
rc18cell2	PC	5.29	8.89	4.77			
rc20cell2	Sub	8.40	10.35	8.18	0.18	3.57	2.98
rc20cell3	CM	10.06	12.63	9.40	0.05	3.51	4.51

Supplementary Table 2. List of all anatomically identified neurons (n = 79) with their record identification (ID) and anatomical location in the thalamus (nucleus). based on the Mouse Brain Atlas [1]. Also shown are physiological parameters for every cell (see Materials and Methods). Blank spaces

indicate absence of data for the particular parameter. Abbreviations: MD. mediodorsal nucleus; PV, paraventricular nucleus; PC, paracentral nucleus; PT, paratenial nucleus; CM, centromedial nucleus; AM, anteromedial nucleus; Rh, rhomboidal nucleus; Re. reuniens nucleus; Sub, submedial nucleus; VM, ventromedial nucleus.

1 Franklin, K. B. J. & Paxinos, G. *The Mouse Brain in Stereotaxic Coordinates*. (Academic Press, 2007).
